# Analysis of Satellite Compass Error’s Spectrum

**DOI:** 10.3390/s20154067

**Published:** 2020-07-22

**Authors:** Andrzej Felski, Krzysztof Jaskólski, Karolina Zwolak, Paweł Piskur

**Affiliations:** 1 Polish Naval Academy, Faculty of Navigation and Naval Weapons, Smidowicza St, 69, 81127 Gdynia, Poland; a.felski@amw.gdynia.pl (A.F.); k.zwolak@amw.gdynia.pl (K.Z.); 2 Polish Naval Academy, Faculty of Mechanical and Electrical Engineering, Smidowicza St, 69, 81127 Gdynia, Poland; p.piskur@amw.gdynia.pl

**Keywords:** satellite compass, accuracy, spectrum analysis, Fourier transform

## Abstract

The satellite compass is one of new variants of satellite navigational devices. Is it still treated with caution on International Convention for the Safety of Life at Sea (SOLAS) vessels, but has become popular on the fishing vessels and pleasure crafts. The standard data obtained by such devices suggest accuracy of satellite compasses at a level of about 1 degree, so it seems to be as accurate as gyro or the magnetic equivalent. A changeability of heading errors, especially its frequency spectrum, is analyzed and presented in the paper. The results of comparison of an onboard standard gyrocompass, a fiber-optic gyrocompass (FOG) and a satellite compass in real shipping circumstances have been discussed based on the available literature and previous research. The similar comportment of these compasses are confirmed, however, in real circumstances it is difficult to separate heading oscillations produced by the ships yaw (or helmsman abilities) from the oscillations of the compass. Analysis of the heading oscillations has been performed based on the measurements of the heading indications of stationary compass devices and the devices mounted on the vehicles moving on the straight line (straight part of a road and tram line) to separate the impact of the vessel steering system. Results of heading changeability in the frequency domain are presented based on the Fourier transform theory.

## 1. Introduction

It is impossible to navigate a vessel without any directional reference. All movements, no matter for people or for vehicles, in environments such as desserts, seas or air, require direction indicators. The contemporary ship is equipped with a magnetic compass and a gyrocompass as indispensable devices. This refers to all the open-sea ships which must be equipped in compliance with the International Convention for the Safety of Life at Sea (SOLAS). Toward the end of the 20th century, satellite compasses began to trace in a completely new way [1,2] in the form of a specific version of a multiantenna GNSS receiver with the additional option to determine a ship’s heading. The most popular is the two-antenna solution, which gives an opportunity to measure two angles: heading and pitch or heading and roll, depending on how it is installed in relation to the centerline of the ship. Three-antenna solutions are also accessible. They allow measuring the full information of the ship’s orientation in the space. According to Sperry Marine [3], one of the manufacturers of such devices, it has been designed as a low-cost alternative to conventional spinning-mass and fiber-optic gyrocompasses for application on workboats, commercial fishing vessels, large private yachts, naval patrol boats, and small merchant ships, which are not required to carry a gyrocompass.

The origin of this devices can be found in Very Long Base Interferometry (VLBI)—a radio-astronomical method in which space sources of electromagnetic signals (usually quasar) are collected by multiple radio telescopes distributed on the Earth [4]. On this basis, by means of correlation of random-type noise registered in the same time in different places (global network), distances between telescopes can be calculated. In the 1960s and 1970s, it was a very efficient method in geodesy, geodynamics etc. on a global scale.

**Figure 1:**
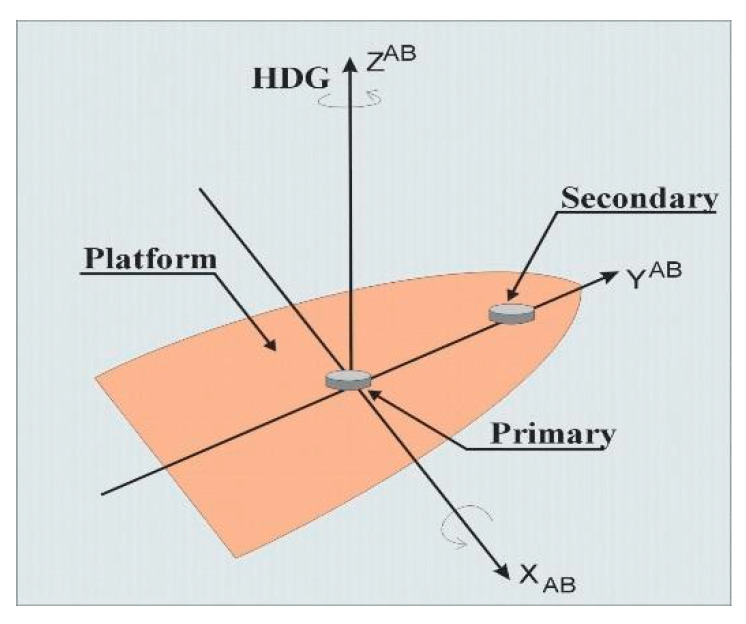
The idea of the use of two antennas to determine the heading of the ship [1].

Signals from satellites can be treated in the same manner. A correlation between signals received by an array of antennas, distributed in a specific way, give us an opportunity to calculate direction on the source of the signal (satellite) when the structure of this signals is known. In the case of a Global Positioning System (GPS) satellite, we are working with a 19 cm long electromagnetic wave, so the distribution of receiving antennas can be of around 1 m. By using two receiving systems and utilizing the carrier wave of GPS signals, we are in fact using (RTK) Real Time Kinematic GPS technology. In the simplest version, the two antennas, namely, base (primary) and rover (secondary), are situated along one of the axis of the ship. In the classical version of RTK, the coordinates of the primary antenna should be known; then, a spatial vector between both antennas can be calculated. As we are not interested in very accurate measurements of the antennas’ positions and the base distance between both antennas is known (due to the fact that a base line between the two antennas is constant and situated in a constant position referring to the hull of the ship), the distances between each antenna and satellite enables us to determine the angles in two axes (Figure 1, axes X and Z).

Such ideas appeared at the end of the 20th century. The numerous publications of Calgary authors, including [5,6,7,8], are particularly noteworthy. Before the manufacturers proposed such devices, many researchers used them as reference systems for compass testing, for example [9,10] 

Proposals for using the GPS system-derived devices to determine the angles of the spatial orientation of the object, using multiple antennas, appeared earlier. Anthony Evans is the author of significant achievements in this area by using the GPS system to determine ship orientation in the 1980s [11]. He proposed a method of measuring orientation angles through a single antenna that cyclically rotates inside the aircraft fuselage. In 1988, he began experimenting with an 18-channel receiver that used a system of three antennas spaced from 40 cm to 60 cm apart. This satellite compass precursor was tested under a marine conditions on the “USS Yorktown” to determine an accuracy during movement [12]. Parameters such as the duration of system initialization, maintaining its continuity and required accuracy in a real time in a dynamic environment, were examined at the beginning of satellite compasses development. Confirmation of the hypothesis was obtained that multi-antenna GPS receivers, in addition to the positioning ability, are able to determine reliable data regarding a ship’s spatial orientation. Another example is the proposal contained in the patent of 1998 [13]. The authors proposed a compass, which determines its spatial orientation based on a construction with two antennas rotated by a stepper motor until a phase equalization of both antennas occurs.

The first devices available to wider users were introduced in the 1990s. In 1991, Ashtech launched the first multi-antenna GPS receiver: 3DF. Using this system, it was possible to determine the heading, longitudinal and transverse tilt, as well as the position, using a system consisting of four antennas, one of which served as the base antenna, and three others were supporting the base. Each antenna cooperated with a separate receiver. All of them, using signals from at least four different satellites simultaneously, by measuring the difference in phase, determined the orientation of the antenna assembly in three-dimensional space. The research was continued by scientists from the University of Calgary, who in 1994 conducted tests on the Canadian research ship, “Endeavor”. Four GPS antennas were mounted on the vessel’s helipad. The purpose of the tests was to compare the indications of this system with the Sperry Mark 3 Model C gyro compass available on the vessel [14,15]. Other analyses related to this subject, especially over the optimal configuration of antennas, have also been published in [5,16].

In 1994, Trimble introduced a four-antenna system called TANS Vector [17]. The system performance was based on phase measurements between one of the antennas (main, base) and each of the others, which were treated as slave antennas.

## 2. Materials and Methods

### 2.1. Background

**Figure 2:**
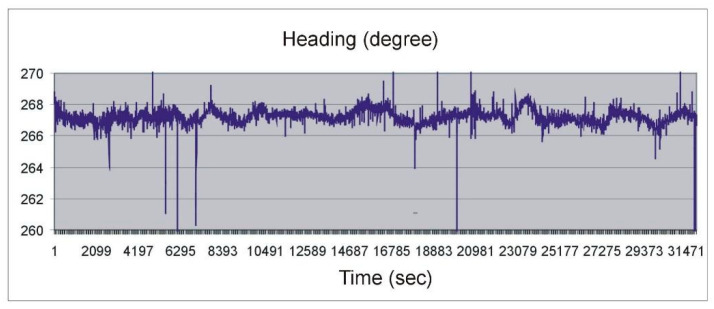
Heading measured with a satellite compass during stationary measurements [2].

Despite many tests confirming the usefulness of multi-antenna GNSS receivers for measurements related to spatial orientation, devices that met the criteria specified in international conventions have been developed no earlier than in 2005. Large errors appeared periodically in all the previous constructions, which were related to the changing satellite constellation parameters. An example of such measurements, taken in 2004 with the Crescent compass installed on the roof of the building is shown in Figure 2.

The MX 575 compass, which as the first receiver of a certificate allowing it to be used as a heading transmitting devices, was introduced in 2005. It could be used as a backup source of heading information in IMO-compliant (International Maritime Organisation) vessels. One of the important solutions was the use of the MEMS-type (Microelectronic Mechanised System) gyroscope, which stabilizes the indications when “raw” measurements in the radio domain turn out to be temporarily inaccurate. Modern constructions often have a triad of gyroscopes and accelerometers. They are integrated systems, able to continue working for several minutes even in the event of satellite signals disappearance. The dynamic properties of such devices largely depend on the details of an algorithm used for the calculation and filtering of signals. The traditional gyro-compass has an electromechanical sensor, whose center of gravity is shifted relative to the geometric center, and thus behaves like a pendulum oscillating with a period of about 84 min (Schuler period, Schuler tuning). Maximilian Schuler made a proposal in 1923 that gyro compasses lend themselves to particularly successful tuning when the curvature of the Earth is taken into consideration. In this way, the instruments can be made insensitive to the disturbances that are caused by the result of the accelerations of the carriers along the surface of the Earth. According to this requirement, the instruments have to be tuned to an oscillation period of 84.3 min. Thus, the classical gyrocompass has its own fluctuation with a long period. There are no kinematic problems in satellite compasses, however, the results depend on changes in the satellite constellation and the properties of the measurement-processing algorithm. This is an issue addressed by the authors of this article. Manufacturers commonly describe the quality of such devices by declaring their accuracy based on an average square error or using similar methods. The assumption of white noise may not be true. Dynamic errors are caused by dynamic factors affecting the system, such as vibrations, roll, pitch or linear acceleration. According to [3] ‘this error may have an amplitude and frequency related to the environmental influences and the parameters of the system itself’. However, for implementation in more complex measuring systems, when the fundamental issue is the selection of devices with different error characteristics, the question regarding the error frequency spectrum is important. The basic principle of integrating devices that perform similar functions is to vary the output error rate.

**Figure 3:**
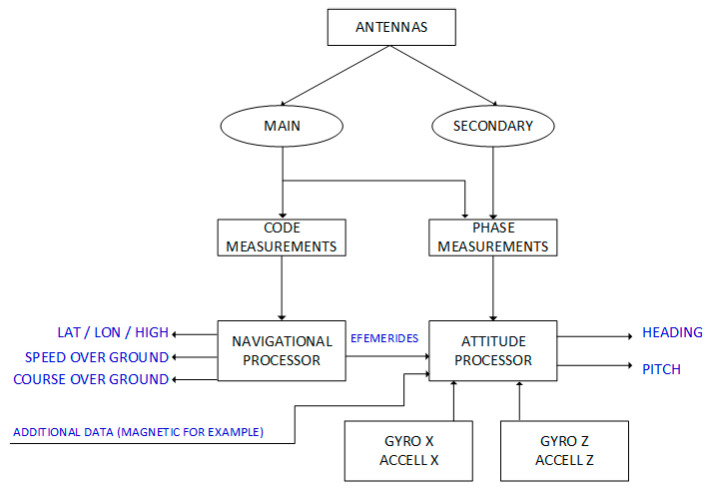
Possible block-diagram of a standard satellite compass. (Source: A.Felski).

There are two main types of satellite compasses available on the market now: dual-antennas and tri-antennas. The most popular are dual-antenna constructions, which give the opportunity to measure two angles in transverse directions to the base between two antennas (pitch or roll in addition to heading). Designs with three antennas give the opportunity to measure all three angles of orientation of the carrier. Besides the number of antennas, there are devices with a constant distribution of antennas and movable antennas, so the distance between them can be changed by the owner or by the fitter. Additional sensors, commonly made in MEMS technology, are used to stabilize angular measurements. In addition to gyroscopes, these devices are often equipped with accelerometers. These are extremely useful for measuring the heave, which is thought to be important on small hydrographic units. In more extensive systems, there is also an option to include information from magnetic sensors or (and) a barometric measuring element. Possible block-diagram of a standard satellite compass was depicted in Figure 3.

**Figure 4:**
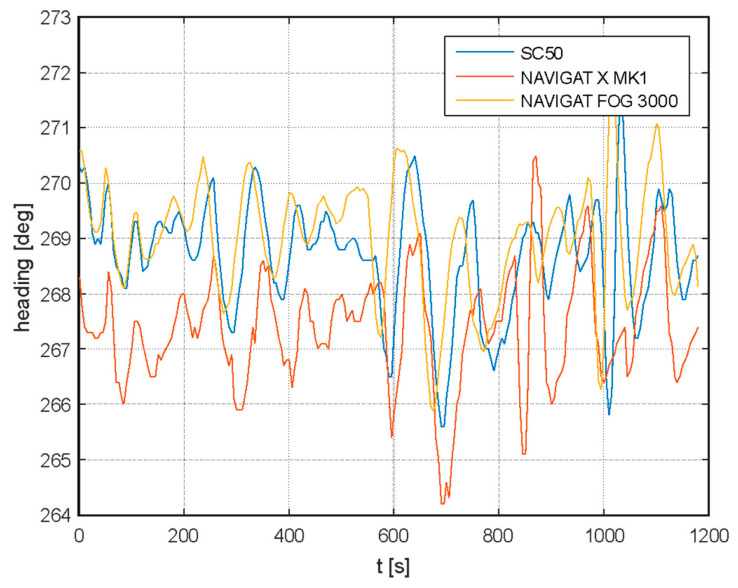
Example of registered headings from a classic gyrocompass (NAVIGAT X) fiber-optic gyro (NAVIGAT FOG 3000) and satellite compass FURUNO SC50 [18].

Studies published in [18] proved that a satellite compass behaves similarly to a standard gyro or fiber-optic gyro (FOG) on a ship in motion. In the Figure 4 a small, systematic shift of measurements from individual devices can be noticed, however, this is due to inaccuracies in the installation of the satellite compass and FOG for the time of experiments. In general, all compasses seem to show almost the same values, and visible oscillations are probably due to imperfections in the control system and inertia of the ship. The existence of a very low frequency (Schuler tuning) characteristic of a classic gyro compass (NAVIGAT X) is noticeable. The most changes in the heading presented in the image occur due the behavior of the ship, so they are very similar to each other.

The spectral analysis of these measurements proves that low-frequency oscillations dominate, and one can also distinguish oscillations common to all three compasses, i.e., yaw resulting from the characteristics of the ships movement (0.01 Hz).

**Figure 5:**
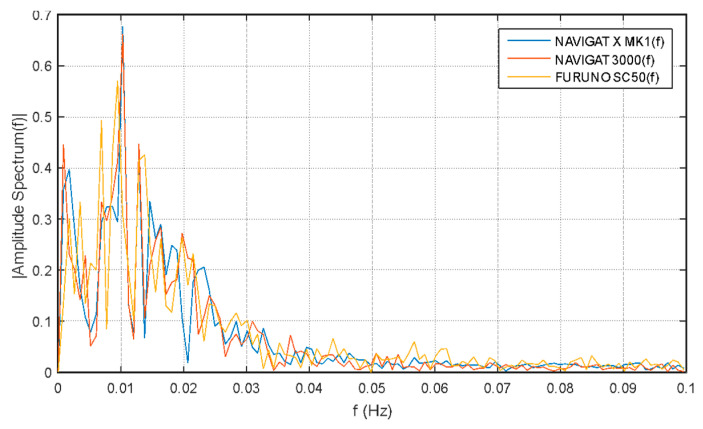
Single-side amplitude spectrum of oscillations presented in Figure 4 [18].

On the other hand, various frequency bands are not clearly repeated in registrations made with individual compasses. For example, significant differences occur at around 0.008 Hz for the satellite compass (as shown in Figure 5).

### 2.2. Devices

The satellite compasses of three different manufacturers were used in the experiments presented in this paper. These are Novatel PwrPak7D, the Advanced Navigation GNSS compass in a low-cost variant and Furuno SC-50. Basic parameters, based on specifications provided by manufacturers, are presented in Table 1.

Novatel PwrPak7D-E1 is a robust GNSS receiver that combines dual antenna signal and (INS) - inertial navigation system hardware in a single enclosure to provide easy-to-deploy industry-leading position and heading data. In this experiments, two G5Ant-4AT1 models made by Antcom were used. According to the manufacturers, the device is suitable for ground vehicle, marine and air-based systems. Its software takes into account SPAN (synchronous position, attitude and navigation) technology based on GNSS+INS sensors as well as ALIGN software for angular determinations. It uses an OEM7720 receiver card and Epson G320N MEMS (IMU) – inertial measurement unit. 

Advanced Navigation is a compact, low weight device, designed for marine and automotive applications, including small-size vehicles. It contains a 9 axis IMU that is integrated with a dual antenna GNSS receiver. Antennas are placed inside a 672 mm enclosure, together with all the signal processing electronic components. This seems to be around 3.25 lengths of the L1 wave of distance between the centers of the antennas. The core of this device is composed of two u-blox M8T GPS modules. The incorporation of all the processing components into the antennas enclosure makes it easy to integrate it even in restricted space conditions. Data are sent through the serial cable or via the (NMEA) National Marine Electronics Association 2000 network. The device is certified to be used on commercial vessels.

Furuno SC-50 is a popular satellite compass for commercial shipping. The large size of its processing unit qualifies it specifically for use on vessels. With its three-fold antenna, it can be useful for surveying ships. Clear display is designed for installation on the vessel’s bridge, although data or data recorders can be sent to other devices using the standard marine format, NMEA 0183, or NMEA 2000 with the proper converters. The SC-303 antenna unit applied in tests consists of three antennas in one robust housing with a 650 mm diameter and a distance between the centers of the antennas of 430 mm. This is about 2.25 of the L1 wave length. 

In summary, three different GPS receivers with different antennas and different distances between them, as well as different algorithms for angular calculations, were tested. The data were acquired with a data recording rate of 1 Hz.

### 2.3. Conducted Experiments

In this paper, two kinds of experiments are reported: stationary and dynamic. During the stationary part of experiments, all the antennas were situated on a building roof or bench in a suburban area. The second part of tests was conducted using an automotive vehicle driven directly out of the urban area; however, the horizon was partially by trees. In addition, we used the part of our previous experiments conducted in Gdańsk on tramway routes and published in [22] when the surprisingly low accuracy of the Furuno compass was observed. We intended to verify how significant the influence of the surroundings was on the observed errors in that investigation.

#### 2.3.1. Twenty-Four-Hour Stationary Measurement Experiment

**Figure 6:**
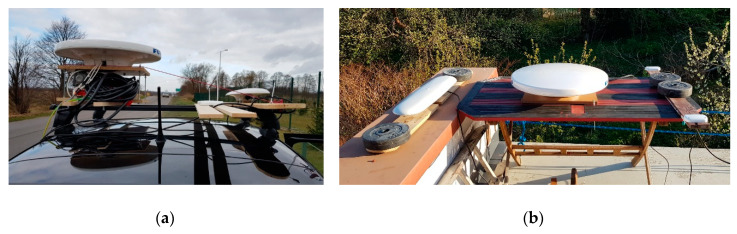
Experimental setup: (**a**) on a car roof for the automotive tests; (**b**) on a house roof for the stationary sets. Photo: K. Zwolak.

**Figure 7:**
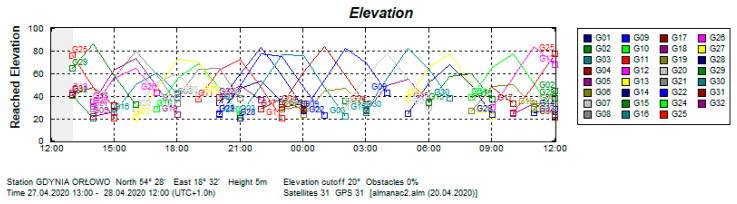
GPS satellite elevations during the experiment on 27–28 April 2020. (Source: Trimble Planning 2.9, 2010).

**Figure 8:**
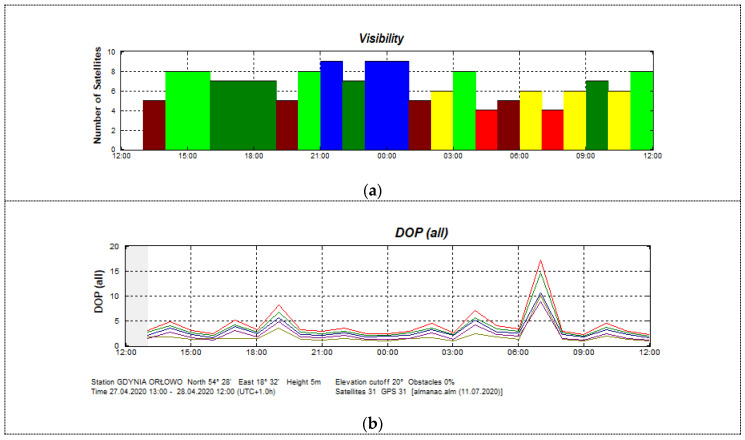
Visibility of satellites (**a**) and dilution of precision coefficients (**b**) in the measurement area on 27–28 April 2020. Notes: Visibility (**a**): red—4 satellites, brown—5 satellites, yellow—6 satellites, dark green—7 satellites, light green—8 satellites, blue—9 satellites. DOP (Dilution of Precision (**b**): red—geometrical DOP, green—position DOP, blue—vertical DOP, brown—horizontal DOP, magenta—time DOP. (Source: Trimble Planning 2.9, 2010).

The stationary experiment results displayed the typical characteristics of this type of test. It was performed from April 27 1300 UTC to April 28 1200 UTC, 2020 in the suburban area of Gdynia, Poland, in the vicinity of a wall of a one-story building, partially obscuring the sky from the north side. The compasses’ antennas were placed 4 m above the ground (Figure 6b). Data were recorded using the NMEA 0183 protocol through the RS-232 and RS-422 serial ports. The satellite constellation was assessed before data registration and the cut off angle of 20 degrees was applied. GPS satellite elevation during the test is presented in Figure 7 and their visibility is presented in Figure 8. The Advanced Navigation compass was set to the stationary variant of measurements. The following settings were applied for Furuno SC50: sampling frequency, 1 Hz; position smooth, 5 s; (SOG) speed over ground smooth, 5 s. A sampling frequency of 1Hz was set for the Novatel compass. 

**Figure 9:**
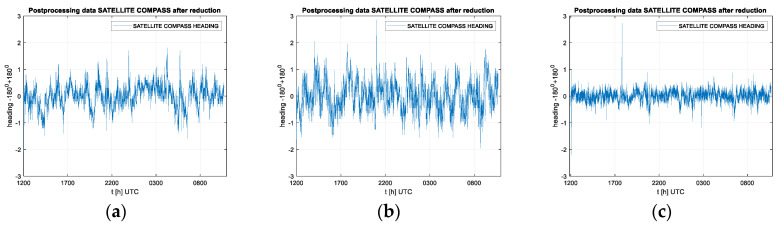
The example subsets of stationary heading registrations by the Advanced Navigation compass (**a**), the Furuno compass (**b**) and the Novatel compass (**c**) (27,28 April 2020).

It must be emphasized that the heading determination needs signals from at least five satellites, in contrast to the position determination, which requires four. Only four satellites have been available twice during the data registration (this occurred at about 0300 UTC and 0600 UTC on April 28). The largest distortions between real time and average heading value in this test were observed for the FURUNO SC50 compass (Figure 9b). The maximum heading distortion for this compass is 2.8 degrees. The offset values fluctuated between −2.0 and +2.8 degrees. Similar results were observed for the Advanced Navigation compass with heading distortion values in the range of -2.2 to +2.7 degrees (Figure 9a). The lowest values of the exchange rate distortion registered for the NOVATEL PWRPAK 7D-E1 compass, however, during the tests, its antennas were 1.2 m apart, which is twice the distance of the other two cases. The heading distortion in this case varied from −0.7 to +0.8 degrees (Figure 9c).

The root mean square of the heading distortion for the Furuno compass is 0.6 degrees, for Advance Navigation is 0.4 degrees and for Novatel is 0.2 degrees.

**Figure 10:**
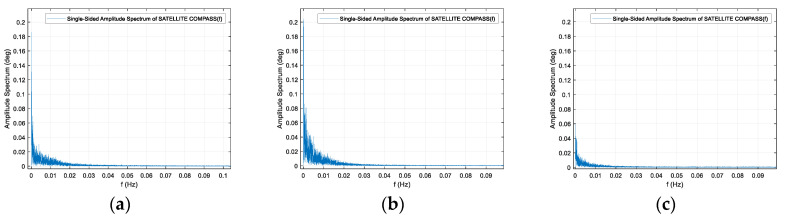
Heading registration spectrum for the three compasses: (**a**) Advanced Navigation, (**b**) Furuno, (**c**) Novatel. Stationary experiment, 27,28 April 2020.

In order to perform the spectrum analysis of the signal in the frequency domain, the presentation of the frequency band in the range above f = 0.1 Hz was abandoned due to the negligible variability of the signal amplitude—heading distortion, which is typical for stationary measurements. Stationary registration results are very similar for all three compasses, characterized by a very low frequency of heading changes, falling in the band lower than 0.02 Hz. However, the amplitudes of these changes vary. The maximum value for the Furuno compass was 0.21 degrees, for Advanced Navigation 0.19 degrees, and for the Novatel product, it was only 0.08 degrees. Undoubtedly, this is due to the length of the base line between antennas, but it can be assumed that this is also the result of the different method of filtration or azimuth calculation. Heading registration spectrum for the three compasses are shown in the Figure 10. 

#### 2.3.2. Automotive Experiments

The analysis of heading oscillations in compass indications in stationary conditions were performed based on calculating distortions from the average value. In this case, the direction of the compass does not matter. In dynamic conditions, the reference direction is needed. Therefore, the tests were conducted in such conditions that the direction of movement of the object was known and determined by natural conditions, i.e., on a straight sections of road or tram track. Knowing the heading of the vehicle during movement, the distortions of individual readings and the oscillations were calculated, treated as corrected measurements, and analyzed using a Fourier analysis. Matlab scripts were written to perform the analysis.

**Figure 11:**
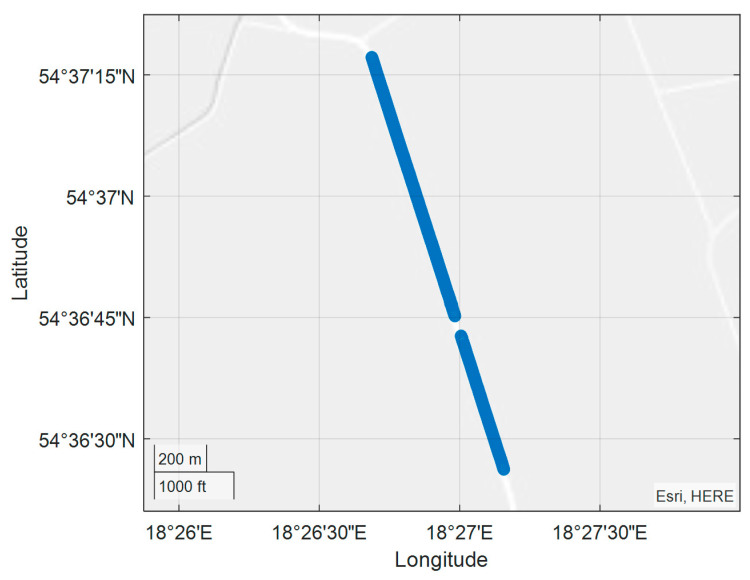
Positions recorded during the road test with the gap in data registration visible on the map view.

Automotive experiments, with the antennas mounted on the roof of the car, were carried out on a straight section of a rural road with a length of 1550 m and a direction of 342/162 degrees. There are single tall trees in the central part of the test section, along the road, and from the east side, which can occasionally cause interference. This is visible in the Figure 11 in the form of a break in position data registration due to incidental obstruction of the satellite signal. Such a gap is a result of a specific configuration of the satellites during this test. During other tests, similar gaps occurred in other places. Unfortunately, it was not possible to guarantee a repetitive configuration of the satellites, however, these records can be treated as examples of how important and diverse the impact of obstructions on the work of such compasses can be. The devices have options to adjust to the vehicle movement, that is, the Novatel compass has “sampling frequency: 1 Hz” and Furuno has “position smooth, 1 s; SOG smooth, 1 s; sampling frequency, 1 Hz”. In addition, it is worth noting that the compasses have advanced inertial systems for the stabilization of readings, but this did not ensure the complete elimination of rapid changes at the time of appearance of another configuration of the satellites received by the device due to the appearance of obstructions.

**Figure 12:**
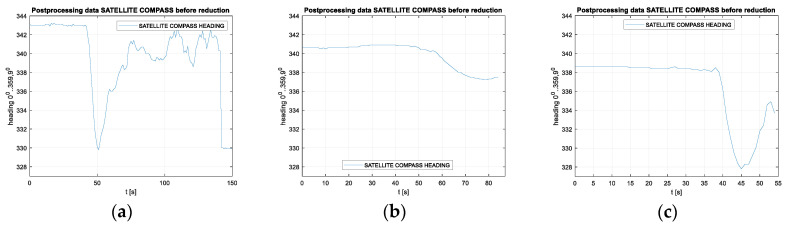
Raw heading records for the Furuno compass for the speeds of 10 km/h (**a**), 20 km/h (**b**) and 30 km/h (**c**).

**Figure 13:**
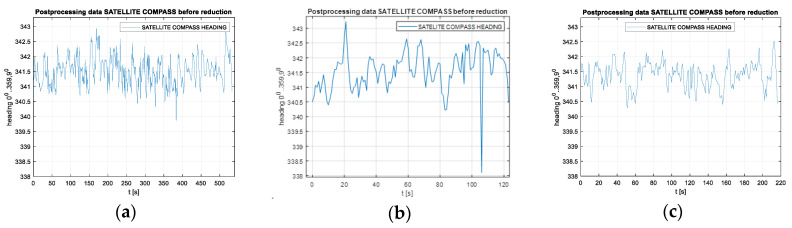
Raw heading records for the Novatel compass for the speeds of 10 km/h (**a**), 20 km/h (**b**) and 30 km/h (**c**).

Measurements were carried out at speeds of 10, 20 and 30 km/h. Raw heading records for the compasses used in this part of the experiment are presented in Figure 12 for the Furuno compass and Figure 13 for the Novatel compass.

**Figure 14:**
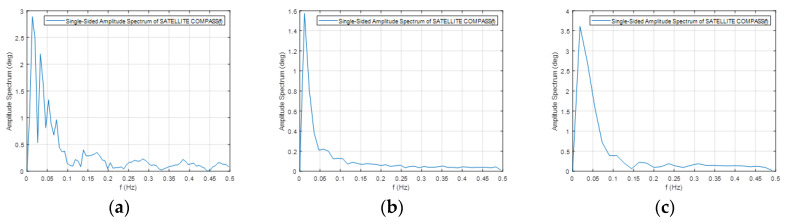
Frequency spectrum for the Furuno compass raw heading records in Figure 12 for the speeds of 10 km/h (**a**), 20 km/h (**b**) and 30 km/h (**c**).

**Figure 15:**
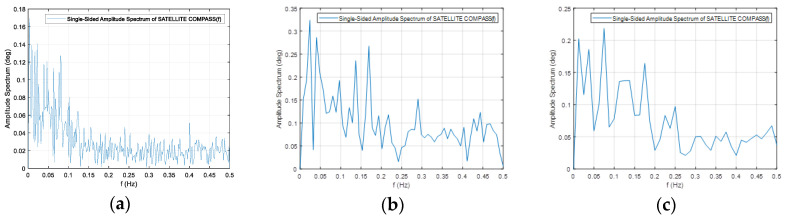
Frequency spectrum for the Novatel compass raw heading records in Figure 12 for the speeds of 10 km/h (**a**), 20 km/h (**b**) and 30 km/h (**c**).

The frequency spectrum of the signals presented above are plotted in Figure 14 and Figure 15.

**Figure 16:**
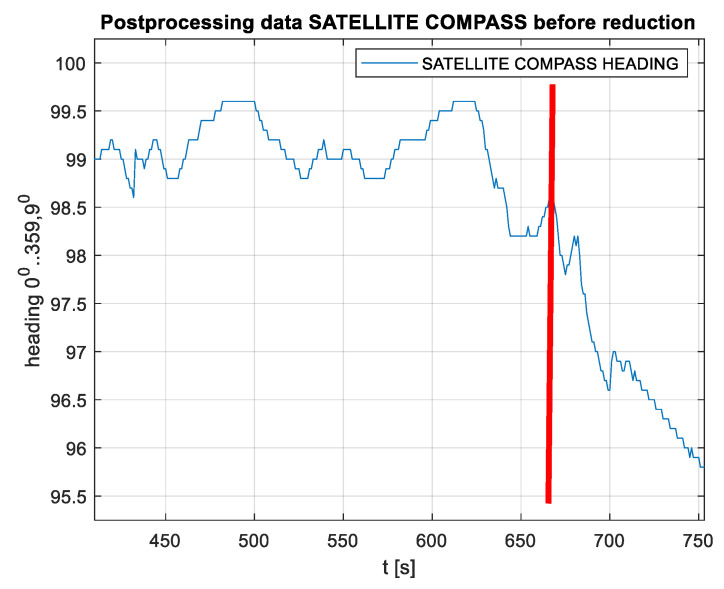
Raw heading value results during the stationary test with the Advance Navigation compass used as an example. The orange line denotes the satellite signals in the moment of being completely obscured.

In the context of a reaction to rapid changes in the satellite constellation, there is also a question regarding the impact on the stability of compass indications based on inertial sensors that are able to support the work of the radio (satellite) segment [23]. An example of the behavior of the Advanced Navigation compass in the case of complete obscuring of satellite signals (667 s after the start of registration) is shown in Figure 16. A clear drift of values is observed, which was similar in other tests, although the directions of the drift were different. After approximately 100 s, no information about the heading was reported by the device.

#### 2.3.3. Tests on Tram Rails

**Figure 17:**
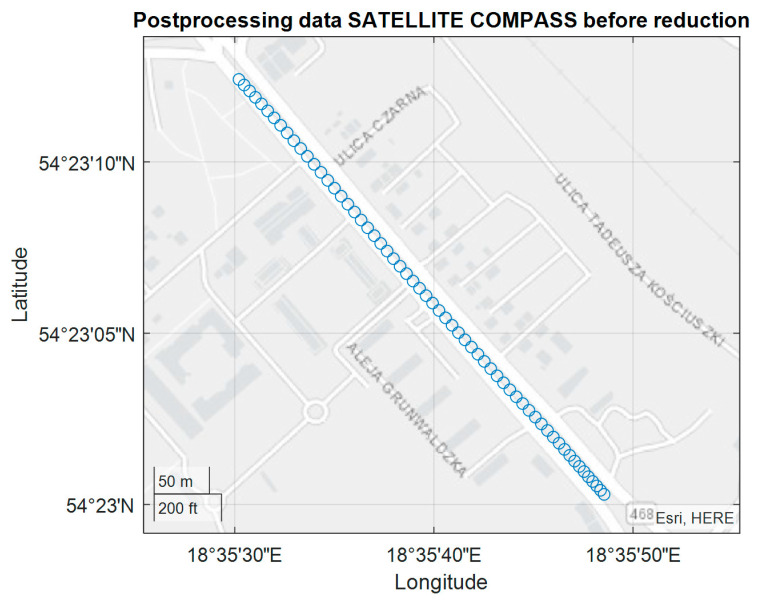
Position registration of the tram from 23:06:25 (UTC) - Universal Time Coordinated to 23:07:25 UTC.

The tram experiment was performed on November 28, 2018 along the route indicated in Figure 17 with the use FURUNO SC50 only. The experiment was conducted in Gdansk on a several-kilometer tram rail with variable sky visibility conditions, with the aim of assessing the performance (accuracy) of the satellite compass operation in non-standard terrain conditions. The measuring instrument used in the experiment was placed on a trolley of the DWF 300 series tram and pulled behind a tram [22] above the tram rails axis. The task of this measurements was more complex, and we now use only a small part of this registration made on the rail part, characterized by a constant direction (Figure 17).

**Figure 18:**
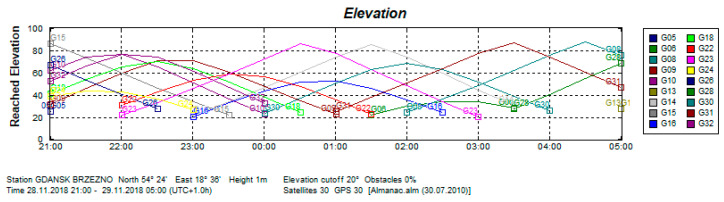
GPS satellite elevations on 28,29 November 2018. (Source: Trimble Planning 2.9, 2010).

For this analysis, it is important that the tram route ran through an urbanized area and some sky obstructions were observed during the registration. The GPS satellite elevations are presented in Figure 18.

**Figure 19:**
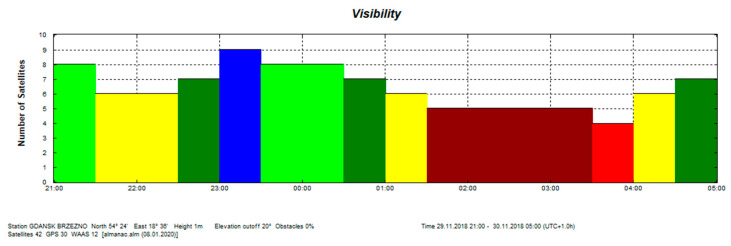
GPS satellite visibility on 28,29 November 2018. (Source: Trimble Planning 2.9, 2010).

The satellite compass requires a signal from at least five satellites for each antenna to determine vehicle heading. Based on Figure 19, it can be seen that a condition of a visibility of at least five satellites to quantify the heading of the vehicle with the arbitrarily assumption of the elevation cut-off at 20 degrees has been met during the experiment.

**Figure 20:**
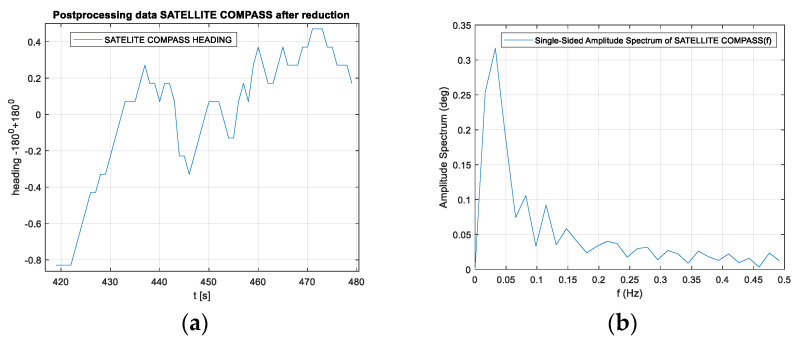
(**a**) Heading distortions. (**b**) Frequency spectrum of the heading records. Heading distortions and the frequency spectrum of the heading records from 23:06:25 to 23:07:25, from the position LAT: 54.386780° N, LON: 018.591723° E to the position LAT: 54.383415° N, LON: 18.5968183° E. (Source: K. Jaskólski).

The presented test was started at 2200 UTC with the visibility of six GPS satellites (Figure 19). The problem occurred when the number of visible satellites was reduced to four (from 0230 UTC to 0300 UTC on November 29, 2018). Based on the data registered from 22:59:27 UTC on 28.11.2018 to 03:26:41 UTC on 29.11.2018, the parts of the straight tram rail section have been chosen. Data have been recorded from the 419th second to 479th second of the run, which is from 23:06:25 to 23:07:25, from the position (LAT) Latitude: 54.386780° N, (LON) Longitude: 018.591723° E to the position LAT: 54.383415° N, LON: 18.5968183° E. Heading oscillations for a vehicle on tram rails were observed in the range of −0.8. to + 0.5 degrees. The values of the heading distortions and the frequency spectrum of the heading record changes are presented in Figure 20.

The heading distortion analysis in the time domain in Figure 20a confirms the declared accuracy of the device indications in accordance with the technical specification of the device, which is 0.51 degrees (RMS) - Root Mean Square error. The spectrum analysis of the signal in the frequency domain in Figure 20b differs from that recorded during the stationary tests because oscillations appear at frequencies higher than 0.02 Hz. The maximum amplitude is slightly higher than that during the stationary record.

**Figure 21:**
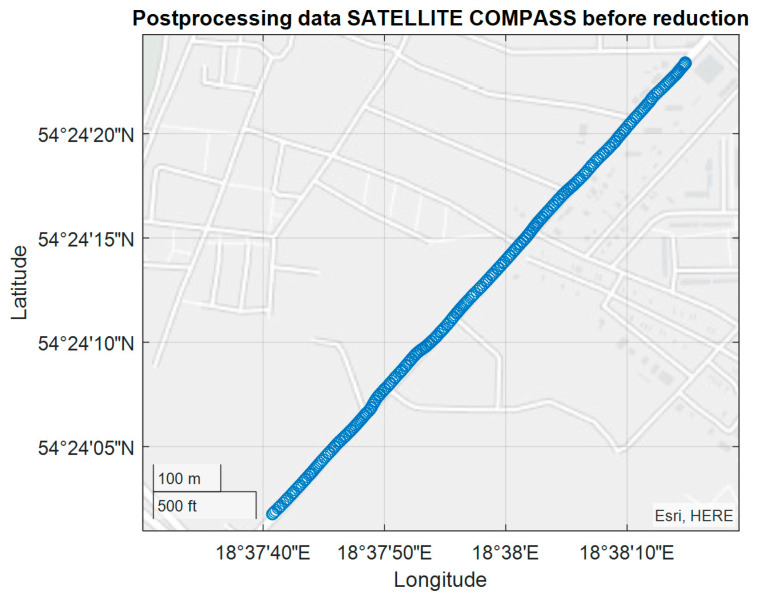
Position registration of the tram from 00:25:17 UTC to 00:30:37 UTC.

**Figure 22:**
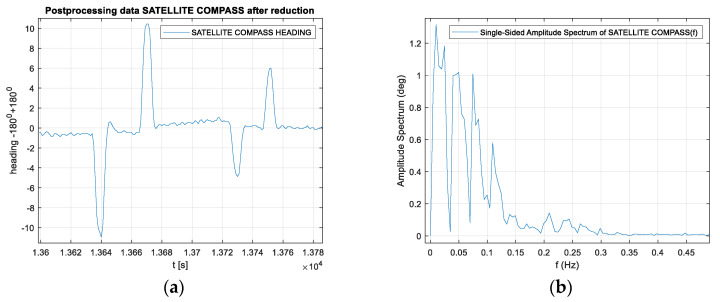
Heading distortions from 00:25:17 UTC to 00:30:37 UTC (**a**) and a frequency spectrum of heading changes (**b**).

Another example of a registration on tram rails is shown in Figure 21 and research scores with a few course deviations are shown in Figure 22. In the heading record, there are four observed significant distortions from the track direction, which result from sky obstructions caused by high buildings in the vicinity of rails. The spectrum of this record differs significantly from others, which is undoubtedly caused by these four clearly distorted parts.

## 3. Results

Studies have confirmed the different accuracies of the devices used in the experiment. It can be assumed that compasses with a fixed antenna system (Furuno and Advanced Navigation), built primarily for seagoing vessels, have an RMS error of approximately 0.5 degrees. The Novatel compass with adjustable distance between the antennas was observed to have higher accuracy, but this is obvious due to the fact that a longer antenna base line (over 1 meter) has been used.

It was confirmed that changes in the constellation of satellites accepted for the solution are the reason for the oscillations occurring in heading registrations. Typical frequencies appearing in the error spectrum are very low, but less than 0.02 Hz for objects in movement. These oscillations are caused by changes in the set of tracked satellites and the satellites included in the calculations. For moving objects, oscillations occur due to changes in the orientation of the vehicle, as well as unpredictable changes occurring as a result of obstructing the satellites by obstacles in the environment.

## 4. Discussion

The motivation to conduct the research was the authors’ experience, published in [18], and especially the results described in the paper [22], in which the results of Furuno compass errors turned out to be surprisingly high. In this paper, the authors were interested in a solution that would be potentially useful on a small floating object used for hydrographic measurements. The issue of the accuracy of the position determined by such a device was purposely not analyzed here, because today it seems to be a trivial issue and comes down to the choice of support service (augmentation) of the GNSS system. This text focuses on the issue of accuracy of the heading, based on the knowledge that the accuracy of the heading measured with each type of compass has different dynamic characteristics. An assessment of a measurement of heading uncertainty on a moving object requires taking into account changes in object orientation angles, because the readings will include both this information and the inaccuracy of the compass or any other gauges. For this reason, three different compasses were tested during static and dynamic experiments. Dynamic experiments, with heading reference data included, were carried out at different speeds on straight sections of tram rails and on straight sections of a roads.

**Figure 23:**
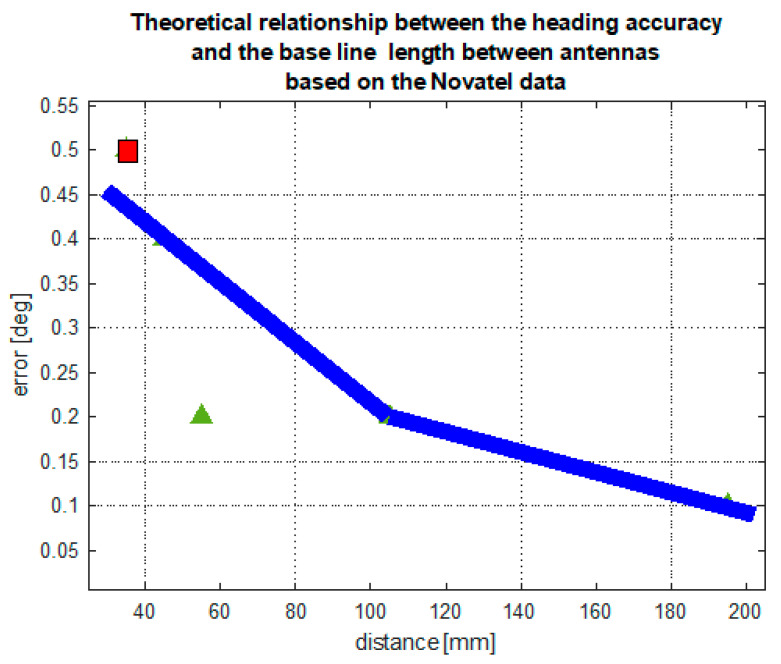
Theoretical relationship between the heading accuracy and the base line length between antennas, based on the Novatel data published in the device documentation (**black curve**). Declared accuracy of the Furuno compass as a function of baseline (**red square**) and of the Advanced Navigation compass (**green triangle**).

The results of stationary experiments confirm the clear relationship between the antenna base line length (distance between antennas) and heading accuracy. Analyzing the compass documentation, this relationship can be presented in a form of a curve, presented in Figure 23. The curve presented here is obtained by interpolating the sparse data regarding the Novatel compass with the quadratic function. The red square represents the manufacturer’s information regarding the Furuno compass, which is consistent with data on Novatel. However, Advance Navigation data deviate from this relationship. The results of stationary tests confirm the declarations of the Novatel and Furuno compass manufacturers, although the Advance Navigation compass tests showed an error of twice the value—0.4 degrees.

For dynamic applications, knowledge of the error spectrum is extremely important. This spectrum is characterized by the dominance of very low frequencies, which is understandable with the use of filtration and inertial sensor support. One can clearly indicate the 0.02 Hz value as the upper limit of these oscillations for all tested compasses. However, in dynamic conditions, higher frequencies appear, which result from slight changes in the orientation of the object during movement. In addition, there are also harmonics, which are in fact the results of rapid deviations of results from previous values. The reason is that despite the solutions using inertial sensors and filtration, these compasses are still sensitive to changes in the temporary constellation of satellites used for calculations. Even during the stationary tests, one can easily identify the relationship between changes in indicated heading values and changes in the satellite constellation. This statement is confirmed by the analysis of the devices’ behavior at the time of signal obstructions. It is always associated with a sharp change in the value at the compass output, although the natural change in the set of satellites seems to have less effect on the results than the short-term screening of one or two satellites, which disappear after a few seconds.

The suppositions that appeared in relation to the results presented in [22], that the accuracy of the entire population of the recorded results do not reflect the whole truth regarding the compass, were also confirmed. It is necessary to take into account the effects of appearing obstructions, and the user should be aware of the effects of the nearby obstructions of the operation of the satellite compass.

The mentioned jumps in the heading measurement for a particular compass cannot be clearly determined with regards to time and amplitude. These changes, although similar and occurring almost at the same time in different compasses, are not identical. Sometimes they are slightly shifted in time for different compasses, relative to the change in constellation. This is clearly seen in Figure 9, where synchronous registrations made in the same place are shown, and sharp changes occur at different times. For example, after 1700 and just before 2200, the sharp changes can be observed in all three registrations at almost the same moments, but their amplitudes differ. There is no rule that one particular compass always shows larger changes. These changes can be considered as a typical filtration effect used in the integrated system. This kind of effect also appears in the coordinate indications referred to by the GNSS receiver, which is a separate element of the tested device. However, the code principle of a position measurement results in fundamental differences from the phase principle of angle measurement. Therefore, during measurements, especially in motion, there were several cases observed in which the heading indications were less accurate or even incorrect, while the coordinate values were correct. Such cases occur when, due to the presence of the obstructions, the configuration of the satellites has decreased to four, while setting the heading requires observation of signals from five satellites. Practitioners using such devices should pay attention to this fact. It is common to observe the values of DOP and (HDOP) – Horizontal Dilution of Precision as indicators of the quality of the receiver’s work, while this refers to the position and is not true in relation to the heading (Figure 8 and Figure 9). It seems that for applications, such as hydrographic vessels, it would be advisable to propose a new indicator to facilitate the control of heading information quality in satellite compasses.

## 5. Conclusions

Satellite compasses have been known for about 20 years and certainly now they can be treated as well-developed solutions. They are becoming increasingly popular in a wide range of applications, including shipping and aviation. They are particularly attractive in controlling self-propelled robots and machines. Their small size and light weight make them very attractive for small autonomous vehicles, except for underwater devices.

At the same time, it is obvious that, like any technical solution, satellite compasses have some limitations. The key is the dependence on satellite signals, which, in the case of the occurrence of terrain obstructions, becomes a significant limitation on land or in inland waters, where the near-shore objects often obstruct satellites. This aspect, however, also appeared recently in the context of potential interference with GNSS signals or spoofing. The literature and media report many cases of interference with satellite signals, even in the sea and in the air. This leads to the question of the legitimacy of the full dependency of navigation systems only on a satellite compass in terms of heading. This aspect is particularly important in the context of planned offshore autonomous vessels, where the risk of interference with satellite signals can cause many complications, not only in the context of the position, but also the heading and effective satellite communication.

When assessing the accuracy of satellite compasses, it should be noted that for several years, they have been constructed as systems supported by inertial sensors (gyroscopes and accelerometers), and now also by other sensors, such as magnetic sensors or those based on pressure. As a result, integrated systems are created, whose measurement properties largely depend on the structure of the data-processing algorithm. This, in turn, takes into account the purpose of the designed system. When assessing a modern satellite compass, one should take into account the branch of applications for which the device was constructed. There are different expectations for the fishing boat and the quadcopter. The requirements may even be different for a ship performing hydrographic measurements in river estuaries, and different for a ship performing similar measurements in the middle of the North Sea or the Gulf of Mexico.

The experiments presented here focused primarily on the spectrum of frequencies that appear in the recorded results of the heading measurements by the mean of satellite compasses. It was confirmed that the reason for the occurring oscillations is the changes in the constellation of satellites accepted for a heading solution. Typical frequencies appearing in the error spectrum are very low, below 0.02 Hz; however, the movement of the object on which they are installed, as well as obstacles causing obscuring satellites result in rapid changes in measurements values, which is evident in the registration of the occurrence of various higher frequencies in the spectrum of heading changes.

For people using such devices, importance should be placed on being aware that the quality of the satellite compass and the quality of the positioning receiver depend on various factors. Therefore, although they are in the same device and many users treat them as one device, one cannot draw conclusions about the quality of the course on the basis of indicators resulting from DOP, which characterize only the positional service. Geomagnetic storms and traveling ionospheric disturbances (TIDs) are also known as sources of GPS positioning quality deterioration. GPS scintillations lead to a range of errors in GNSS due to diffraction [24]. Deep signal fades that appear during small-scale irregularities effect the result in navigation outages [25,26]. Satellite compasses are very popular in high-latitude regions, however, the aurora borealis becomes especially visible in such regions, and the effect of the Earth’s ionosphere on GNSS signal propagation (total electron content) is one of the main error sources which limits the accuracy and reliability of GNSS applications [27].

## Figures and Tables

**Table 1:** Basic technical parameters of the three compared satellite compasses used in the experiments [19,20,21].

No.	Feature	Novatel PwrPak7D-E1	Advanced Navigation	Furuno SC-50
1.	Position accuracy	0.6 m (DGNSS)	2.0 m or 0.6 m – (DGNSS)	3–10 m (depending on corrections)
2.	Velocity accuracy	0.03 m/s	0.05 m/s	no data
3.	Roll and pitch accuracy	no data	0.4°	no data
4.	Heading accuracy	0.1° (for 2 m base length)	0.2° (base length 620 mm)	0.5° RMS (base length 430.3 mm)
5.	Heave accuracy	no data	5% or 0.05 m (whichever is greater)	no data
6.	Output data rate	Up to 100Hz	Up to 100 Hz	Up to 40 Hz
7.	Base length	adjustable	permanent 620 mm	permanent 430.4 mm
8.	Supported navigation systems	GPS L1, L2	GPS L1 SBAS GALILEO E1 BeiDou B1	GPS L1

Where: (GPS) Global Position System, (SBAS) Satellite Based Augmentation System, L1, L2 - frequency of GPS, (RMS) Root Mean Square, DGNSS - Differential Global Navigation Satellite System.
